# Trajectories of Activities of Daily Living in the Last Eight Weeks of Life Among Patients With Terminal Cancer in a Palliative Care Unit: A Retrospective Study

**DOI:** 10.1089/pmr.2023.0043

**Published:** 2024-02-02

**Authors:** Ryo Soeda, Aiko Ishikawa, Shunsuke Oyamada, Mana Mitsuhashi, Suzune Okano, Aiko Yokosawa, Teruo Okutsu, Tetsuya Tsuji

**Affiliations:** ^1^ Department of Rehabilitation, Tsurumaki-Onsen Hospital, Hadano, Kanagawa, Japan.; ^2^ Department of Rehabilitation Medicine, Keio University Graduate School, Shinjuku, Tokyo, Japan.; ^3^ Department of Rehabilitation Medicine, Keio University School of Medicine, Shinjuku, Tokyo, Japan.; ^4^ Department of Biostatistics, Japanese Organisation for Research and Treatment of Cancer, Arakawa, Tokyo, Japan.; ^5^ Mominoki Day Care Centers, Yamato, Japan.; ^6^ Okuda Clinic, Hadano, Japan.

**Keywords:** activities of daily living, cancer, palliative care, terminal care

## Abstract

**Background:**

Although cancer patients' activities of daily living (ADL) are reported to decline before death, ADL trajectories have not been sufficiently clarified due to limitations in the assessment and analysis methods.

**Objectives:**

To clarify the multiple trajectories of ADL in patients with terminal cancer using a comprehensive assessment measure.

**Design:**

This was a retrospective observational study.

**Setting/Study Subjects:**

Cancer patients aged ≥18 years discharged at death from a single-center palliative care unit.

**Measurements:**

Functional Independence Measure (FIM) total scores for eight weeks retrospectively.

**Results:**

In total, 306 patients were analyzed. Group-based trajectory modeling analysis estimated four groups as the best model for the FIM trajectory over eight weeks using the following trajectories: (1) a No Decline group, in which ADL did not decline until just before death; (2) a Rapid Decline group, in which ADL declined rapidly two weeks before death from a trajectory similar to the No Decline group; (3) a Moderate Disability and Slow Decline group, in which the patient slowly declined from requiring mild-to-severe assistance; and (4) a Severe Disability group, in which the patient continuously required severe assistance.

**Conclusions:**

Multiple ADL trajectories were identified in the last eight weeks of life of patients with terminal cancer. These findings suggest that palliative care needs to be tailored to the characteristics of each patient.

## Introduction

Understanding the disabling process at the end of life is essential for informed decision-making among patients with cancer, their families, and medical professionals. One-third of patients with cancer are reported to have difficulties or need assistance with activities of daily living (ADL),^1^ which professionals should support. ADL represents one of the main factors comprising a good death, and maintaining the ability to carry out ADL is important for a better end of life.^2,3^

Previous studies have reported that the ADL of patients with terminal cancer declines rapidly from 12 to 4 weeks before death,^4,5^ resulting in most patients requiring full assistance at one week before death.^5,6^ However, the ADL assessments in these studies did not use measures that detected gradual changes in the level of assistance, and thus could not detect patients with a gradual decline in ADL.^4,6^ Additionally, attempting to identify the trajectory of ADL by means or median values might not properly detect several patterns of decline in ADL before death because only one trajectory can be analyzed.^4–6^

Group-based trajectory modeling (GBTM) is a semiparametric method used to identify different subgroups of individuals who have shown similar patterns of change over time for a given variable. This method can potentially reveal trajectory groups other than those with a rapid decline before death among patients with cancer. Yasui et al.^7^ analyzed ADL trajectories using GBTM in 22 patients with cancer, but it was difficult to interpret the results because of the small number of cases.^8^ Gill et al.^9^ examined ADL in 383 community-dwelling older adults at one year before death using GBTM, but the subjects included a variety of noncancer diseases, so the trajectory of patients with cancer remains unclear.

Therefore, this study aimed to evaluate ADL using the Functional Independence Measure (FIM),^10^ which enables the identification of gradual changes, and GBTM analysis to clarify the trajectory group of patients with terminal cancer.

## Materials and Methods

### Study design and study participants

This study was designed as a retrospective cohort study. Data were collected from electronic medical records in the medical database of the Rehabilitation Department of Tsurumaki-Onsen Hospital in Kanagawa, Japan. The patients in this palliative care unit are mostly those with terminal cancer. Patients are admitted for symptom relief and end-of-life care.

The study participants were patients with cancer who had been discharged from the palliative care unit of Tsurumaki-Onsen Hospital between April 1, 2015, and August 31, 2019. The inclusion criteria were as follows: (1) patients who died during hospitalization in the palliative care unit, (2) patients aged 18 years or older, and (3) patients with at least one FIM assessment. Patients were excluded if they were hospitalized for more than 13 weeks and were discharged from the palliative care unit to a hospital, home, or other institution.

Patients hospitalized for more than 13 weeks were excluded because they were not considered to have the same characteristics as other patients in terms of social background (e.g., living alone). Because variables related to medical conditions were unavailable for this study, only deceased patients were included and changes in their ADL were analyzed based on the date of death.

This study was conducted according to the Declaration of Helsinki. Approval was obtained from the Tsurumaki-Onsen Hospital Clinical Research Ethics Review Subcommittee (approval number 396). Because the subjects were deceased, the opportunity to opt-out was guaranteed to the bereaved family and other concerned stakeholders.

### Primary outcome

The FIM is used to assess ADL. The FIM is composed of 13 motor and 5 cognitive items.^10^ Each item is scored on a seven-point scale, from 7 (complete independence) to 1 (total assistance), with total scores ranging from 18 to 126. Although each item on the FIM is an ordinal scale, the total score is treated as an interval scale because Rasch analysis has confirmed that the scale is unidimensional.^11^ The palliative care unit at our facility conducted an FIM evaluation of the patients at admission and every two weeks thereafter to understand and share information about their ADL and consider the rehabilitation and nursing care plans. This FIM evaluation was performed as part of routine clinical care by a rehabilitation professional, with nursing evaluation as needed.

The study used FIM data for the eight weeks before death that were retrospectively available from the patient's medical records. The data for the eight weeks before death were included in the analysis, with the assessment immediately before death set as week 0. The eight-week data were expected to reveal a trajectory group other than those who showed sudden decline at four weeks before death similar to that observed in previous studies.^4,5^ If the patient died less than eight weeks after admission, FIM data were not available for all eight weeks, but were used for the number of times the patient was able to be evaluated.

### Secondary outcome

Information on the primary tumor and metastasis examined on admission by the physician was collected retrospectively from the patients' medical records. We also used the revised Tokuhashi score to predict life expectancy in patients with metastatic cancer of the spine.^12^ The revised Tokuhashi score is based on the patient's general condition, number of extraspinal metastatic foci and metastases in the vertebral body, metastases to other internal organs, primary site of malignancy, and the presence of palsy.

### Background factors and medical information

Information on the patients' age, gender, diagnosis, presence of metastasis (e.g., brain, bone, liver, lung), and duration of stay in the palliative care unit were also collected from the patients' medical records.

### Statistical analysis

We estimated the trajectory groups and analyzed the associated factors using the groups as variables. We used GBTM to estimate the FIM trajectory groups in patients. GBTM uses the maximum likelihood method and PROC TRAJ in SAS (SAS Institute, Inc., Cary, NC, USA) to fit a semiparametric mixture model to the longitudinal data.^8,13^ We based GBTM on previous studies.^8,13–15^ Because the study participants were hospitalized for varying lengths of time, the number of FIM assessments for each also varied. GBTM can supplement these missing data; therefore, we could adapt the data of patients with different durations of hospitalization in this study to reduce the risk of bias in the participants' characteristics.

First, a censored normal distribution method was applied, and estimation models were generated for groups 1 to 6. In each model, we determined whether each trajectory was the best fit by linear, quadratic, or cubic terms, referring to the output estimates and *p*-values. Second, we compared the Bayesian information criterion (BIC) to determine the optimal number of trajectory groups; a higher BIC value indicates a better fit, so the model that showed the largest BIC from the output results was considered the best-fitting model. We further evaluated the validity of the final model using the average posterior probability (AvePP). Estimating the model outputs the probability of membership in each group. The AvePP is the average of the probabilities that an individual is most likely to belong to a specific group. Previous studies recommended that it should be greater than 0.7;^8,15^ therefore, we adopted the same criterion in the present study.

We used multinomial logistic regression analysis to analyze the factors associated with each of the estimated trajectory groups and the backgrounds of the patients. The estimated trajectory group was the dependent variable. Gender, age >65 years, presence of metastasis to the brain, bone, liver, and lung, and the revised Tokuhashi score^12^ (type of primary tumor) were used as the independent variables.

The statistical significance threshold was set at 5%. SAS 9.3 (SAS Institute, Inc.) was used for the GBTM and multinomial logistic regression analysis, and SPSS version 25.0 software for MAC OS (SPSS, Inc., Chicago, IL, USA) was used for the descriptive statistics.

## Results

### Demographics of the study participants

In total, 306 patients (162 males [52.9%], 144 females [47.1%]; mean age, 76.73 years) who met the eligibility criteria from among 416 who had been discharged from the palliative care unit of Tsurumaki-Onsen Hospital were included in the study and evaluated in terms of the FIM over time. Of these patients, 54 were discharged home, 1 was transferred to another hospital, and 38 were hospitalized for more than 13 weeks; 17 patients who did not have an FIM evaluation were excluded. Among all patients, 134 (43.8%) were hospitalized from home, 18 (5.9%) from institutions, 15 (4.9%) from convalescent hospitals, and 139 (45.4%) from hospitals at which their cancer had been treated. The median duration of hospitalization was 23 days. Table 1 shows the patients' primary site of cancer and classification of distant metastasis. In total, 120 (39.2%) patients were without metastasis; the rest had one or more metastases. Single metastasis was the most prevalent (*n* = 109 [35.6%]), and metastasis to three sites was the least prevalent (*n* = 18 [5.9%]). The revised Tokuhashi score was 0 for 139 patients (45.4%), 1 for 30 (9.8%), 2 for 57 (18.6%), 3 for 25 (8.2%), 4 for 34 (11.1%), and 5 for 21 (6.9%). All patients belonged to the “poor” prognostic group.

**Table 1.** Primary Cancer Site and Number of Metastases

Primary cancer site	***n*** (%)
Brain	6 (2)
Head and neck	26 (8.5)
Lung	66 (21.6)
Breast	11 (3.6)
Gastrointestinal tract	71 (23.2)
Liver/gallbladder/pancreas	55 (18)
Urological	20 (6.5)
Prostate	8 (2.6)
Gynecological	27 (8.8)
Skin	3 (1)
Blood/lymph	5 (1.6)
Bone/soft tissue	5 (1.6)
Unknown	3 (1)
Total	306 (100)

Metastasis site	***n*** (%)
Brain	38 (12.4)
Bone	71 (23.2)
Lung	92 (30.1)
Liver	80 (26.1)

The number of patients evaluated at each time point was 41 (13.4%) at eight weeks before death, 75 (24.5%) at six weeks, 126 (41.2%) at four weeks, 207 (67.6%) at two weeks, and 306 (100%) at just before death.

### FIM trajectory and related factors in the eight weeks before death

**FIG. 1.**
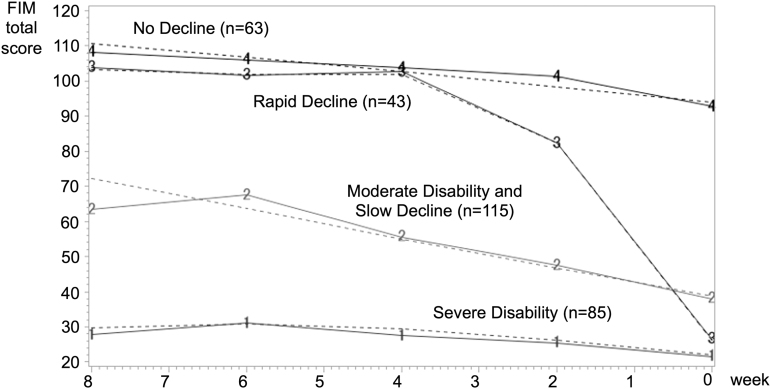
FIM trajectory groups at eight weeks before death as estimated by GBTM. The solid line shows the mean value and the dotted line shows the estimated value. From the top of the figure, the No Decline group (No. 4 on the solid line), the Rapid Decline group (No. 3 on the solid line), the Moderate Disability group (No. 2 on the solid line), and the Severe Disability group (No. 1 on the solid line). FIM, Functional Independence Measure; GBTM, group-based trajectory modeling.

Four groups were selected as the best model based on the output results (Appendix Table A1). The GBTM analysis estimated the following four trajectories (Fig. 1): (1) a No Decline group, with a total FIM of 111 points at eight weeks before death and 94 points just before death, which is still nearly independent; (2) a Rapid Decline group, with a total FIM of ∼100 points from eight to four weeks before death with the same trajectory as the No Decline group, but with a decrease to 82 points at two weeks before death, and a further decrease to 26 points at just before death indicating the need for severe assistance; (3) a Moderate Disability and Slow Decline group, in which the patient already required moderate assistance at eight weeks before death (total FIM of 72 points), and then slowly decreased to 39 points at just before death indicating the need for severe assistance; and (4) a Severe Disability group, with a total FIM of 30 points at eight weeks before death and 22 points at just before death, indicating the need for persistent severe assistance. The percentage of patients belonging to each trajectory was 20.9% (*n* = 63) in the No Decline group, 18.8% (*n* = 43) in the Rapid Decline group, 34.3% (*n* = 115) in the Moderate Decline group, and 26.1% (*n* = 85) in the Severe Disability group.

Multinomial logistic regression analysis was performed with the No Decline group as the reference, the trajectory group as the dependent variable, and patient background as the independent variable (Table 2). In the Severe Disability group, brain metastasis (odds ratio [OR]: 10.3, 95% confidence interval [CI]: 2.6–40.5, *p* < 0.001), lung metastasis (OR: 0.36, 95% CI: 0.17–0.80, *p* = 0.03), age >65 years (OR: 2.93, 95% CI: 1.11–7.72, *p* = 0.03), and bone metastasis (OR: 2.93, 95% CI: 1.28–6.74, *p* = 0.01) were extracted from the Moderate Disability and Slow Decline group. No significant differences were found for the other independent variables.

**Table 2.** Multinominal Logistic Regression Analysis

	Group (No Decline group as the reference)	OR	95% CI	***p***
Male	Severe Disability group	0.81	0.40–1.62	0.54
	Moderate Disability and Slow Decline group	0.80	0.42–1.53	0.50
	Rapid Decline group	0.46	0.20–1.03	0.06
Brain metastasis	Severe Disability group	10.30	2.60–40.5	<0.001^*^
	Moderate Disability and Slow Decline group	3.31	0.83–13.2	0.09
	Rapid Decline group	2.42	0.43–13.8	0.32
Bone metastasis	Severe Disability group	2.32	0.94–5.73	0.07
	Moderate Disability and Slow Decline group	2.93	1.28–6.74	0.01^*^
	Rapid Decline group	0.47	0.12–1.87	0.28
Lung metastasis	Severe Disability group	0.36	0.17–0.80	0.03^*^
	Moderate Disability and Slow Decline	0.64	0.32–1.27	0.20
	Rapid Decline group	0.61	0.25–1.46	0.26
Liver metastasis	Severe Disability group	0.82	0.35–1.91	0.65
	Moderate Disability and Slow Decline group	1.47	0.71–3.07	0.30
	Rapid Decline group	1.91	0.79–4.63	0.15
Age >65 years	Severe Disability group	2.93	1.11–7.72	0.03^*^
	Moderate Disability and Slow Decline group	2.34	0.98–5.60	0.06
	Rapid Decline group	1.54	0.54–4.45	0.42
Tokuhashi score	Severe Disability group	1.03	0.83–1.27	0.80
	Moderate Disability and Slow Decline group	0.99	0.82–1.21	0.97
	Rapid Decline group	1.03	0.80–1.32	0.82

The factors of each group were analyzed with reference to the No Decline group. Metastasis was analyzed with reference to the No Metastasis group.

^*^ *p* < 0.05.

CI, confidence interval; OR, odds ratio.

## Discussion

### ADL trajectory of patients with terminal cancer

Previous studies have reported that the ADL of patients with terminal cancer declines from one month before death, with severe to full assistance in one week to a few days.^4–6^ Lunney et al.^4^ reported changes in ADL among cancer patients, with a mean number of ADL items requiring assistance of 0.77 at one year before death, but a mean of 4.0 at four weeks before death. Seow et al.^5^ reported that palliative performance status decreased from an average of 54.7% (frequent assistance level) at four weeks before death to 41.3% (mostly assistance level) at one week before death. McCarthy et al.^6^ assessed ADL (bathing, dressing, urinary control, transfers, toileting, eating, and walking) in patients with rectal cancer and nonsmall cell lung cancer at 24 weeks before death. The results revealed five ADL impairments from one month to three days before death and seven at three days before death. The assessment measures used in previous studies have included items other than ADL, such as the oral intake of food and level of consciousness^5^ or only assessed whether independence was possible.^4,6^ In addition, a gradual decline in ADL cannot be detected when assessed as independent or not, which may result in a sudden decline in ADL when the patient requires supervision or assistance. Furthermore, those previous studies showed a single trajectory by mean or by median. For these reasons, a comprehensive and gradual assessment of ADL was needed to clarify the current condition of ADL in patients with terminal cancer, in addition to a method that reveals multiple trajectories.

In this study, the FIM was used as a measure to detect patients who maintained their ADL immediately before death and those whose ADL declined during the eight weeks before death.^10^ The GBTM analysis of the FIM data enabled us to identify multiple groups of trajectories. ADL assessment by the FIM and the GBTM analysis allowed us to identify patient independence and the amount of graded assistance, successfully demonstrating the trajectory of the four groups.

### Factors associated with the trajectories

Multinomial logistic regression analysis revealed that the factors most strongly associated with Severe Disability group (No Decline group as reference) were brain metastasis (OR: 10.3), age >65 years (OR: 2.93), and lung metastasis (OR: 0.36) (Table 2). Neurological disability and decreased ADL have been reported in patients with metastatic or primary brain tumors.^16,17^ At the end of life, these patients have symptoms such as unconsciousness, drowsiness, and cognitive impairment.^18,19^ These patients are also reported to have more nursing problems with ADL compared with other patients with cancer.^20^ Therefore, patients with cancer with brain metastases are assumed to require more severe assistance with ADL because of cognitive and conscious impairments. On the other hand, the median age of the patients in this study was 77 years, and most subjects were aged >65 years. Costantini et al.^21^ studied ADL in patients with cancer in the 52 weeks before death and reported that they tended to be lower in patients aged 65–84 and ≥85 years than in those aged 18–64 years. We consider that older age is a potential factor for lower ADL and identified this as a factor related to the Severe Disability group. In contrast, lung metastasis was less related to the Severe Disability group. Although few reports have investigated the effect of lung metastasis on ADL, Yoshioka^22^ reported no significant difference in the Barthel Mobility Index between patients with and without lung metastasis. Patients with lung cancer often experience dyspnea,^23^ which may affect mobility. However, unlike brain or bone metastases, lung metastases do not directly cause central nervous system or musculoskeletal impairment. Therefore, we believe the association with the Severe Disability group is lower.

Multinomial logistic regression analysis showed that the factor most strongly associated with Moderate Disability and Slow Decline group (No Decline group as reference) was bone metastases (OR: 2.93) (Table 2). Patients with bone metastases have been reported to have decreased ADL compared with those without.^22^ The pain, pathological fractures, spinal cord compression symptoms, and hypercalcemia that can occur with bone metastases are referred to as skeletal-related events (SREs).^24^ When SREs occur and cause pain at rest and during movement and motor and sensory paralysis in the extremities, the ability to sit, stand, and walk is impaired, and ADL are reduced.^25,26^ However, bone metastases frequently metastasize to the spine, pelvis, and femur,^26^ which enables patients to use their upper limbs in bed or a wheelchair, and some activities, such as eating and dressing, may not require assistance. Therefore, bone metastases was identified as a factor associated with Moderate Disability and Slow Decline.

### Limitations

This study had several limitations. First, it was conducted at a palliative care unit. In hospitals, environmental factors, such as the room size and corridor distance, differ from those at home, and patients may receive more assistance than necessary to prioritize safety and help prevent falls. Therefore, the patients in this study may have had less independence in carrying out ADL.

Second, because the FIM is assessed every two weeks after admission, the assessment just before death (0 weeks) can range from 1 to 13 days. Therefore, ADL are expected to decline further during this period. In particular, the No Decline group may have experienced a rapid decline in ADL during this period. We cannot deny the possibility of a decline in ADL that was not revealed because of the range of assessment periods.

Third, the Tokuhashi score was used for cancer sites, but site-specific studies are needed. In addition, the existence of metastasis was investigated, but because the size of metastasis is considered to differ, the quality of metastasis and its symptoms also need to be evaluated.

Fourth, rehabilitation was prescribed at Tsurumaki-Onsen Hospital. As rehabilitation may influence the maintenance and improvement of ADL,^27,22^ future research should include data on rehabilitation prescriptions and trends in ADL.

## Conclusions

The ADL trajectory group involving patients with terminal cancer who died and had been discharged from the palliative care unit for eight weeks before death was analyzed using GBTM. The results revealed the existence of four different trajectory groups and associated factors. Patients aged >65 years or who had brain metastases were more likely to require severe assistance at eight weeks before death. By contrast, patients with bone metastases were more likely to require mild-to-moderate assistance. Therefore, support plans and interventions considering the trajectory group and its associated factors are necessary for ADL support for patients with terminal cancer. These results could help clarify the multiple ADL trajectories of patients with cancer before death and the associated factors, thereby providing essential knowledge for examining the medical and nursing care services required for patients with terminal cancer.

## Abbreviations Used

ADL: activities of daily living
AvePP: average posterior probability
BIC: Bayesian information criterion
CI: confidence interval
FIM: Functional Independence Measure
GBTM: group-based trajectory modeling
OR: odds ratio
SRE: skeletal-related event

## Appendix

**Appendix Table A1.** Comparison of Group-Based Trajectory Modeling Models by Number of Groups

Group no.	BIC	2ΔBIC	AvePP
1	–3304.83		
2	–3172.85	263.96	All groups >70%
3	–3119.31	107.08	All groups >70%
4	–3074.48	86.66	All groups >70%
5	–3064.48	19.66	Group 2 < 70% and Other groups >70
6	No comparison because there was a group with 0% belonging		

AvePP, average posterior probability; BIC, Bayesian information criterion.
